# A System of Practical Medicine, Comprised in a Series of Original Dissertations

**Published:** 1840-11-07

**Authors:** 


### MEDICAL EXAMINER.

DEVOTED TO MEDICINE, SURGERY, AND THE COLLATERAL SCIENCES.

No. 45.] PHILADELPHIA, SATURDAY, NOVEMBER 7, 1840. [Vol. III.

### BIBLIOGRAPHICAL NOTICE.

*A System of Practical Medicine, comprised in a series of Original Dissertations.* Arranged and edited by ALEXANDER TWEEDIE, M.D., F.R.S., Fellow of the Royal College of Physicians, Physician to the London Fever Hospital, and to the Foundling Hospital, etc. With Notes and Additions by W. W. GERHARD, M. D. Philadelphia: Lea & Blanchard. 1840.

THIS forms the second volume of the Library of Practical Medicine, edited by Dr. Tweedie. The object of the work is to present a view of practical medicine, bringing the subject to the modern times, and including the various additions which have of late years been made to the pathology of disease.

The second volume relates to the diseases of the nervous system, which is a subject offering many difficulties, and embarrassing from the imperfect classification of many of the diseases. Some writers have admitted the symptoms as the only sure basis, and others have confined themselves too exclusively to the pathological lesions: the former has been the error of the English, the latter of the French writers. The author of the articles upon this subject in the Library of Medicine, has not followed either classification to the exclusion of the other, but he has inclined rather to the English than to the French system; and if we were restricted to one of these courses we should incline with the author most to that which lays the greatest stress upon the symptoms.

The treatment recommended by the author of the articles is, in general, very sound and unexceptionable: one article, however, is very defective—that relating to delirium tremens: the additions of the American editor will, it is hoped, supply the deficiency. An addition of some length is also made to the chapter of acute hydrocephalus: if any important deficiencies appeared in the rest of the work, they were supplied by a note. But the general character of the articles is such that few alterations seemed necessary.

We extract the article on Delirium Tremens, because it includes some observations on the treatment of this disease, which will, it is believed, be of importance to the American practitioner. Most of our readers are aware that the ordinary treatment of this disease is by the use of opium in large quantities. This method is successful to a certain extent, but requires great care, and there is no doubt that in many complicated cases it is positively injurious: this is particularly the case in complications of delirium tremens with pneumonia, or with cerebral inflammation. After a number of successive trials the opium practice was abandoned entirely in the management of the cases which form the subject of the succeeding remarks, and the disease was treated in all bad cases, and in many slighter ones, by alcoholic stimulants; the result was entirely successful.

In slight cases, or even in those which were moderately severe, this treatment was not always employed; many of the patients whose cases are included in the summary were treated by vegetable emetics, as lobelia and ipecacuanha, or by antispasmodics, as Hoffman's anodyne, and assafœtida. Others were managed by the simple arterial stimulants, such as capsicum, or were left simply to nature, but the alcoholic practice was relied upon in all bad cases which seemed grave from the beginning, or in which other remedies had failed.

The following remarks, which were added by the American editor to the work in question, will more fully explain the results of the treatment:

Delirium tremens is still a common disease in the United States, although the improvement in the habits of the people is gradually rendering it less frequent. The cheapness of spirits, and the comparative pecuniary comfort of our population, place the means of indulgence in intoxicating drinks within the reach of all classes, and render delirium tremens a much more frequent disease than it is in countries where either distilled spirits are difficult to procure, or the habits of the people prevent their indulgence in them. It is for this reason that the disease has attracted so much attention amongst the physicians of this country. In common with others I have been obliged to make it a subject of investigation, and my position as a medical attendant upon the principal institutions of Philadelphia for a period of nine years, afforded me an extensive field for the study of the disease, and has enabled me to settle some points of practice which have had an important influence on the mortality of the disease.

The distinction generally admitted by writers as to the origin of the disease, is a correct one: there are two distinct modes of origin;—in one, which is the more simple form, the disease begins after complete abstinence from spirits, and in the other the patient continues his habits of intemperance until the disorder breaks out, or until some derangement of an important organ produces an attack of disease which prevents him from indulging longer in his habitual beverage.

1. *Common Delirium Tremens.*—This variety appears a few days, or even the next day, after a fit of drinking, whether it has been carried to the extent of a decided debauch or not, or it may occur in individuals who are simply tipplers, that is, accustomed to drink from two to six or eight glasses of spirits daily, or from a gill to a pint. The patients who are affected with this disorder in private practice, belong for the most part to the latter class, and are taken with the disease when ill health or a surgical injury has suddenly obliged them to give up their habitual stimulants. The attack is generally more mild in those who are accustomed to this less degree of stimulation, than when it directly follows a fit of intemperance.

The disorder is usually divided by authors into three stages, which are not always clearly separated one from another. The distinction is, however, perfectly well founded, and the symptoms of each period are more definite in this disease than in most others. The division is extremely convenient, for the prognosis and treatment of the disorder are extremely different in its several stages, and a disease which is almost always curable at the beginning, becomes in the advanced stage extremely intractable.

(*a*) *First Stage.* This is well known amongst drunkards as the *horrors:* a term which expresses the aspect of the patient, which is that of extreme anxiety and agitation, and the distressing feelings of fear which the patient experiences. This anxious alarming expression, is one of the most characteristic symptoms of the disease, and with the tremor, which is equally remarkable, it constitutes the only pathognomonic character. The tremor extends to the whole muscular system, but as it may be to a certain extent restrained by a voluntary effort of the will, or by supporting the weaker muscles of the limbs against the trunk, it is sometimes not very obvious unless the patient is directed to put out his tongue, or to hold up his hands, where it is at once perceived. The restlessness and tremor are the most frequent and important symptoms of the first stage of the disorder, but are by no means the only ones; the others, however, are only accessary or secondary, and vary with each patient. As a general rule, the pulse is feeble and frequent, the mind is unable to direct itself long to any single subject, and the pupils are slightly contracted. The complexion is extremely variable; it is often pale if the patient has not been long addicted to intemperance, but in the majority of cases it retains the usual tint of the drunkard's countenance. The appetite fails, the bowels are often constipated, and there is generally more or less thirst. In this stage of the complaint, the restlessness continues throughout the night, and of course the patient is unable to sleep: sometimes, the sleeplesness is the first symptom of the disease, but in the majority of cases it attends the restlesness, and is strictly proportioned to it. The agitation may gradually subside and the patient recover, or the disease may pass into the next stage.

(*b*) The *second stage* of the complaint presents the same symptoms as the first, but in an exaggerated degree, the tremors, restlessness, and insomnia are increased, and the appetite is more completely destroyed. The pupils are more contracted; if, however, the patient has not taken opium, the contraction of the pupils is never very great. The distinctive symptom of the second stage, is the illusions which at first occur only at night, when the patient is left alone, and in the dark. These hallucinations are perfectly under the control of the understanding when the courage of the patient is revived by light and society; he is then perfectly aware of their nature, and will often laugh at his own fancies. The illusions are not confined to the night, if this stage become more confirmed, but they still remain perfectly under the control of the will and of the intelligence; if the disease continue, the illusions become more and more frequent, and cease to be recognised by the patient, that is, they are completely confounded with real objects. The attention may still be directed to surrounding objects, and the patient is capable of answering ordinary questions with perfect correctness, if he is addressed in a sharp decided tone of voice, and there is no incoherence in his answers, so long as his attention can be commanded. These illusions are nearly always of an alarming kind, and are as varied in their nature, as the objects which happen to be most familiar to the patient; devils, guns, fire, serpents, and the like, are the most common objects of his fear. At other times he feels a vague dread that his life will be taken, and earnestly entreats that it may be spared. These illusions are so well characterised, that they have always been regarded as the essential character of true delirium tremens; this is nearly, but not absolutely correct, for in some cases the tremors are not attended with illusions, but on the contrary, the mind of the patient is almost clear, and the disease may prove fatal, although no illusions present themselves, by the occurrence of convulsions or sudden insensibility. Still in the regular simple variety, of which I am now treating, the illusions may be regarded as a constant symptom. The other symptoms of the second stage are not pathognomonic, and, with the exception of the countenance, which retains the same restless expression as in the first stage, are not even characteristic. The pulse is frequent, and generally small, the frequency evidently depends rather upon the extreme agitation of the patient than any regular connection between the state of the circulation and the disease. The appetite rarely returns during this stage, although this is sometimes the case; the tongue is generally furred, but rarely dry. The skin remains moist throughout this stage, and if the efforts of the patient to escape from confinement be constant, or if his agitation be very great, the sweat is often very profuse. This sweat is of a different character from that which generally occurs during the third stage of the disorder, and seems to be strictly dependent upon the constant exercise which the agitation of the patient obliges him to take. The second stage may gradually decline, and the patient fall asleep, and recover; or it may pass into the next stage. Sleep is nothing but the indication of the recovery; it follows rather than precedes the decline of symptoms. The insomnia arises from the extreme nervous disturbance which is the essential element of the disease, and although the fatigue of the patient may be extreme, he is still altogether unable to sleep. Let the nervous agitation be quieted by any means, and sleep will immediately follow, and will finally complete the restoration. This is the true rationale of the close connection between sleep and recovery, which has certainly been misunderstood, and has led to erroneous deductions as to the treatment of the disease. If the disease be completely removed, the patient will sleep for a long time, and will generally awake perfectly restored. In some cases, however, the recovery after prolonged sleep is not complete, but the disease recurs again, and is not completely cured until a day or two afterwards. If the prolonged sleep occur naturally, it is always productive of great relief to the patient, but if it be forced by the operation of narcotics in large doses, instead of conducing to recovery, it will sometimes end fatally, and the patient may then die without awaking. A short sleep of one, two, or three hours is refreshing, but is not usually followed by immediate recovery, although it affords an evidence of the gradual decline of the disease. If delirium tremens be well treated, or if the disease be essentially mild, but few cases pass beyond the second stage; recovery taking place without difficulty.

(*c*) The *third stage* is attended, like the others, with a symptom which is characteristic; that is, incoherence. The illusions either cease, or they are no longer connected, the patient passing from one object to the other with great rapidity, and not reasoning correctly or connectedly upon the images which are presented to his mind. He becomes feeble, but is, at the same time, extremely agitated, and can only be retained in bed by the constant watchfulness of an attendant, or by straps or bandages. The sweat becomes profuse, the skin sometimes cold, at others warm, and pupils greatly contracted. The contraction sometimes ceases before death, and may be succeeded by a morbid dilatation if there be much serous effusion upon the brain. The senses become gradually more and more obtuse, from the first appearance of incoherence; the patient generally loses his power of attention, and can with great difficulty be induced to direct his attention to surrounding objects, and as the disease advances, he becomes completely comatose, and generally lies in a state of insensibility for some time before death. The pulse gradually fails during this period, and the patient often presents symptoms of nervous disturbance, which are very analagous to those which take place in cases of typhus fever, such as subsultus, spasmodic tremors of the muscles generally, and muttering delirium.

By adopting this division, and basing the distinctive characters of the different stages, upon the mental disturbance which occurs in each of them, a clearer idea may be formed of the character of the disease. If the third stage is reached, the chances of recovery, of course, are extremely small. But the disease will sometimes terminate fatally without going thus far; those cases which I have seen of this sudden termination, were, for the most part, treated by high doses of opium; I have also seen it occur where little or no active remedies had been given, very soon after the entrance of a patient into a hospital, and after the emetic practice. The sudden termination of the disease is not therefore dependent upon any uniform method of practice. These sudden deaths are more frequent in the second variety, when they are generally preceded by convulsions; these, however, sometimes occur in the milder form.

2. The *second variety* of delirium tremens occurs during a debauch, and patients actually intoxicated are often admitted into the Philadelphia hospital, labouring under this disease. The individuals who suffer with this form, are attacked with it after very high stimulation. That is, the stimulation is extremely great, either because the quantity of spirits is very large, or the patient is extremely susceptible of the effects of alcohol. There is in this variety of the disease, a singular union of the directly exciting action of alcohol upon the brain, with the debilitating effects of a diminished stimulation. The apparent contradiction is in many cases easily accounted for; the stomach in these patients is gradually enfeebled and will not receive, or at least retain the accustomed quantity of spirit, and the nervous tremor therefore supervenes: if the stomach becomes again capable of acting upon the alcohol, the tremors are not arrested, although positive intoxication is sometimes brought on. The disorder consists then of two distinct parts; one is the ordinary form of delirium tremens, which is but slightly modified, and the other is the vascular congestion or inflammation which is directly caused by the alcoholic excitement. If there is simply congestion, the face is flushed, and the eyes often injected, but the colour is of a dark and not a bright red tint, the intelligence is dull, and the whole countenance indicates nearly an apoplectic stupidity. These cases are extremely apt to terminate in convulsive fits, which sometimes do not differ from ordinary apoplexy of the simple congestive form, and in other cases resemble an epileptic attack much more closely than apoplexy. If the fits are frequently renewed, the disease will either terminate fatally in the paroxysm, or coma may come on more slowly, and the disorder merely assume the form of the third stage of delirium tremens. When convulsions take place, the ordinary course of the disease is often singularly modified, and its stages greatly shortened. If the excitement be of a more active inflammatory form, the delirium will assume the characters of ordinary meningitis, and the symptoms of the delirium tremens be gradually merged in them; or the reverse may take place, and the inflammatory symptoms abate and be followed by those of this disease. This variety is neither as frequent nor as dangerous as the congestive form. It must not be confounded with true meningitis or cerebritis, which occasionally occurs in drunkards, especially those who have been exposed to the ordinary causes of cerebral inflammation, as blows on the head received during their debauch, or the heat of a summer sun. This mistake can scarcely take place with those who are accustomed to the disease, but it might occur with practitioners who are not familiar with delirium tremens.

*Complications.*—It is evident, that delirium tremens is a well characterised disease, with its own peculiar symptoms, but it may present numerous complications. These may be either acute or chronic disorders of the viscera. The fevers, properly speaking, rarely complicate delirium tremens, unless they are of the milder forms, such as the ordinary intermittent or remittent, for a severe continued fever comes on gradually, and either prevents the formation of delirium tremens, or it replaces the symptoms by its own peculiar phenomena. The ordinary complications are the vascular congestions or inflammations. These follow the prevailing epidemic constitution of the season, or are caused directly by the action of the alcohol. Thus in summer, dysentery and diarrhœa are extremely frequent, while in winter pneumonia is a common and a very grave complication. In a few cases gangrene of the lungs has occurred during the course of the disease; this was particularly frequent in the winter of 1828—29, when I observed a large number of cases of gangrene at the Philadelphia Hospital. The complications which arise directly from the alcoholic stimulation, are cerebral inflammation or congestions; and gastric disorders, either inflammatory, or the reverse; and various affections of the liver, especially the fatty enlargement. Individuals labouring under phthisis or other chronic diseases, especially hypertrophy of the heart, are sometimes the subjects of delirium tremens. These complications do not in general obscure the symptoms of delirium tremens, except those disorders connected with the brain. The latter occur for the most part in the second form of the disease.

*Diagnosis.*—The diagnosis of the disease is formed from the previous habits of the patient, as well as his present symptoms, and is in general easy; it becomes difficult only when a cerebral disorder more or less similar to delirium tremens occurs in drunkards. The distinguishing characters are the tremors, the peculiar and changing character of the illusions; the sweats and the restless alarmed countenance. The pulse and the other secondary symptoms of the disease are of little value for the diagnosis. The accidental inflammations are readily distinguished by one who is accustomed carefully to examine the condition of the viscera, otherwise they may often escape notice. This is especially the case with the thoracic inflammations, which are singularly obscure in all cases in which the brain is much disturbed, for the functional signs of these affections then cease or become very slight.

*Prognosis.*—The prognosis of the disease depends greatly upon the treatment. If it be treated according to the plan which is here presented, it is almost invariably favourable. Should fatal cases occur, they may be ascribed to some unforseen cause, or some accidental complication which is not to be looked for in the ordinary course of the disease. If the disorder be left to itself, mild cases wear themselves out and terminate favourably, although the suffering of the patient is far from inconsiderable; severe cases pass into the advanced stages of the disease, and are apt to terminate fatally. Other methods of treatment modify the prognosis. The disease is, therefore, much more variable in this respect than those disorders which are less amenable to therapeutics.

*Anatomical characters.*—These are not necessarily connected with those of either congestion or inflammation. The brain may be in either of these states but it is as a purely accidental complication. The most frequent anatomical character is an unusual moistness of the whole brain, serum seems diffused through it, and follows every cut of the scalpel, and is at the same time effused beneath the arachnoid, and contained in undue proportion in the ventricles. Even this appearance I do not regard as necessarily connected with the disease, but I am disposed to refer it, in part at least, to the slow approach of death, and to the laboured condition of both circulation and respiration, which generally precedes it. If this view be correct, the disease must be classed amongst those purely functional disorders of the nervous system which are connected with no regular anatomical lesion; this opinion is confirmed by the history of the symptoms.

*Treatment.*—Of the various other remedies employed in the treatment of delirium tremens, opiates have probably received the most attention. I formerly used these remedies in almost every case, though not in as large doses as some of my brethren; but when I was a resident physician at the Philadelphia hospital, we were directed to give opium in very large doses—frequently as much as four grains every two or three hours, until sleep was procured. The patients, for the most part, got well under this treatment; but in estimating the value of a particular plan of treatment, we ought to consider the proportional success of this and other plans. A comparison of this sort will prove that opium is not the most effectual remedy in delirium tremens. In conjunction with this remedy, certain hygienic regulations were also enforced at the time to which I have alluded. The patients were locked up in cells, and if very disorderly, that is in every severe case, they were confined in a straight jacket, or retained in bed with gloves and straps.

The practice of the hospital has never been to give opium to the exclusion of other remedies; it was always the custom to use cups and cold applications to the head, purgatives and various other remedies, when they seemed necessary. From time to time a change would be made in the practice, and the affection would either be treated upon empirical grounds, or in accordance with the varying symptoms, or the emetic practice would be pursued.

But the plan of treatment by opiates and confinement is the one that was almost universally practised in Philadelphia several years ago, with variable results. In my own practice I have gradually diminished the quantity of opium which I formerly gave, and for some time past have not used it at all. Instead of it, I have relied upon the stimulant treatment which is followed in some parts of New England, and from time to time has been much resorted to in the Philadelphia hospital; that is, the use of stimulating remedies, particularly alcoholic liquors. These articles I first employed in conjunction with opium, or prescribed them without opiates, in two different conditions; 1st, in the slighter cases, or those of incipient delirium tremens; or, 2dly, in the severe cases where opium had been exhibited but was followed by distress of mind and stupor. But at present I use them singly. This treatment has diminished the mortality of the disease, and rendered it almost always curable. The change which I have adopted in the hygienic rules, has also contributed very decidedly to this result. Instead of confining the patients, I let them walk about and enjoy the company of others as much as they choose: merely taking care that some one should be near them to prevent accidents. I was led to this change by observing that the hallucinations which attend the disorder were more distressing when the patients were in a state of confinement than when they were allowed to walk about as much as they wished. As I have already remarked, they are capable of controlling these hallucinations, until the intellect is entirely powerless; and they can do so the more easily when they are surrounded by objects which may serve to engage their attention. Confinement always irritates them, and increases their ravings, so that the third stage, in which the intellect is completely destroyed, is apt to be brought on more speedily. I have very often tested this by a simple experiment; a man who was confined to his bed by a straight jacket, or something of the kind, I have frequently directed to be dressed, have soothed him by conversation, and after requiring a promise that he would conduct himself with propriety, I have very seldom found reason to be dissatisfied with the result. On the contrary, the disease would almost invariably become milder, and the necessity of confinement cease. It is true that confinement is often necessary at night, from the impossibility of always providing a sufficient number of attendants. I therefore (with the exception just stated) allow the patient to have full liberty, the only restraint being the presence of the keeper: sometimes also I direct them to be set at work, which serves still further to distract their attention.

The proportional mortality under the two plans of treatment which I have detailed, is represented in the following summary, comprising the number of cases treated amongst the men for the space of 5½ years—that is, from the 20th of May, 1834, to the 13th of November, 1839. The whole number of cases admitted for delirium tremens, or intemperance, which was expected to terminate in delirium tremens, was 1241. Of these there were 1198 whites, and only 43 men of colour. Of the whole number, 708 were decided cases of delirium tremens, 60 were slight cases, and 430 cases of mere intemperance. Of the latter, some terminated in decided delirium tremens, and others proved fatal from diseases (such as pneumonia) contracted during the fit of drunkenness, for which they had been sent to the lunatic asylum. So that this class furnishes a considerable number of bad cases. Of the whole number, 121 cases proved fatal; that is, a fraction less than one in ten.

In the first year, from May, 1834, to May, 1835, the number of admissions was 141; of these, 18 died; that is, rather more than one in eight. In the second year the number of cases was 211, the deaths 24, or a little more than one in nine. The third year, in 301 cases there were 47 deaths, a much larger proportion than in preceding years, one in 6 19-47ths, but depending upon an accidental cause; that is, the occurrence of an epidemic of typhus, which attacked many of the debauched subjects of intemperance: some of them were sent to the lunatic asylum as labouring merely under the effects of intemperance, and could not be afterwards removed to the proper ward.

In the fourth year, beginning May, 1837, of 206 cases, 19 only proved fatal, that is, about one in eleven. This was a decided amelioration, and coincides precisely with the epoch at which the change of practice was introduced.

In the fifth year the mortality went on diminishing, and was less than one in twenty-six; or of 274 cases, 9 only were fatal; and amongst these cases, the mortality was certainly greatest in those which were treated chiefly according to the method formerly pursued at the hospital.

Finally, in the months ending November, 1839, the mortality was only one in 33¾, that is, 4 cases out of 135; and of these four, one entered moribund, and was not, therefore, treated in the hospital; another had inflicted upon himself several fractures and other injuries, by leaping from a third story window, in a fit of delirium tremens, previously to his entrance. The others, it is believed, were also complicated cases.

The preceding summary of the results of the treatment, is extracted from a lecture which I delivered at the Philadelphia Hospital, in December, 1839. The results of the treatment for the last year, up to the present time, (October, 1840,) have been still more satisfactory. The number of cases of the sequelæ of intoxication, and of delirium tremens in the three stages, admitted into the men's wards of the Philadelphia Hospital, from October 12, 1839, to October 12, 1840, is 223. Of these, 61 were classed under the head of intoxication or its immediate sequelæ, some of them passing into delirium tremens. If we exclude the whole of these 61 cases, there remain 162 cases of decided delirium tremens; of these, 87 were admitted in the first stage, 73 in the second, and 2 in the third; 160 cases recovered, and one remained convalescent, who is since well. (Oct. 16.) One only proved fatal: this patient was admitted in the third stage of the disease, and died in a few hours after his entrance; he had been treated with opium, and a box of pills which he was taking were sent to the hospital with him. Of course, this apparent exception confirms the general conclusion, that the disease terminates favourably in every instance, when treated according to the method recommended.

The proof must, therefore, be conclusive if all the circumstances surrounding the patient remain the same. These have remained precisely as they formerly were, with the exception of the difference in the management and treatment of the patients. The superintendant is the same, the resident physicians, in whose immediate charge the patients remain, are of the same average education and experience, and the other circumstances connected with the disease remain unchanged. The inference is, therefore, rigorously deduced, that the former method of treatment yielded an average mortality, which varied but little in different years; while the treatment now pursued, is followed by a mortality which may be regarded as a mere cypher. The single fatal case which has occurred amongst the list of 162 patients admitted, depending on other causes, and the progressive decline of the proportionate mortality keeping pace with the change of the treatment. If, therefore, evidence of this nature be rejected, or if the facts which were not observed by one person alone, but by a large number, or if a practice which was not carried out by a single resident physician, but by a succession of a large number, be rejected as wanting due confirmation, it is very clear, that the common rules of observation, and the conclusions which, under the ordinary circumstances, would be regarded as beyond cavil, must fail when applied to medicine. This, of course, involves a contradiction that few would be willing to admit, at least to avow.

The treatment substituted for the former practice was conducted according to the following general plan:—If a patient entered in a state of intoxication, whether he was labouring under the early symptoms of delirium tremens or not, an emetic was always prescribed; the best for this purpose is either a simple diluent drink, such as chamomile tea, or warm water, or a dose of ipecacuanha. After the operation of the emetic, the patient was generally tranquil for a time, and sometimes fell asleep. On his awaking, the symptoms of delirium tremens presented themselves, if his debauch had been protracted. If it had lasted only for a few days, the disturbance of the nervous system was limited to a slight agitation, or tremors; many such cases occur and terminate in a day or two. If the disease pass into regular delirium tremens, the treatment does not differ from that pursued in those cases, in which the disease is developed previously to the admission of the patient.

The object is then to remove the disorder of the nervous system which follows the excessive use of ardent spirits, by a milder excitement, which may gradually terminate in recovery. For this purpose the best remedy is alcohol, that is, some form of distilled spirits; in our hospital that employed is the cheaper kind of brandy. Of this, an ounce may be given every three or four hours, if the case be a slight one; if the tremors are more severe, or if the disease is advanced to the second stage, two ounces should be given every four hours, or one ounce once in every two hours. It is very rarely necessary to exceed this quantity in cases which are brought under treatment during the first, or early in the second stage. There are some cases which require, for a short time, larger doses: there are those in which the disease is either of itself more severe, or in which the patient has been in the habit of using enormous quantities of alcoholic stimulus. It is then often necessary to administer the brandy in doses of two ounces every two hours, but these doses are rarely required for more than a single day. In a few cases where the depression is extreme, it becomes necessary to increase the dose even beyond this amount. In two instances at least, the quantity was not less than two ounces every hour for three or four doses; these were, however, extreme cases, of a class which I have never known to recover under ordinary methods of treatment.

The rationale of this practice is evident enough; the excessive stimulation to which these patients have been long subjected is not only followed by a subsequent depression, but is attended with an extreme disorder of the nervous system, which constitutes the essential character of the disease. The stimulating practice relieves this irritability for a time by substituting its own peculiar action, and when administered in these doses, which are never sufficient to intoxicate, the subsequent depression may be completely avoided. Of course, no practitioner who feels a proper regard for his patient, or for his own character, would allow the patient or his friends to increase the quantity of alcoholic stimulants to such a degree as to run the slightest risk of producing these intoxicating effects. The quantity must vary according to the susceptibility of the patient; if this has been nearly destroyed by excesses, the quantity of alcohol which is necessary to produce a given effect must of course be greater than in those cases, in which it is still but little impaired. The dose should always be as small as possible, for if the quantity necessary to tranquilise the patient be exceeded, it acts as an irritant, and produces injurious consequences.

I am perfectly aware that the alcoholic stimulants are not absolutely necessary for the majority of cases of delirium tremens; a variety of methods will cure the disease, or it will in the greater number of cases get well of itself, like all diseases which have a self-limited duration. I wish merely to state what is incontestibly proved by the documents, that the stimulant practice offers a successful result, which may be looked for with a certainty which is almost absolute, and that this method of treatment has the advantage of being applicable to the worst cases; in the milder ones the only question is, whether it unites the advantages of curing the patient "safely, quickly, and agreeably;" of this no one who has witnessed the horrible sufferings of the patients who labour under delirium tremens, and their speedy alleviation by this treatment, can entertain a doubt. The difficulty which will arise in the minds of many, is of a different kind: many physicians will hesitate on moral considerations, from a dread of either seeming to give countenance to the habit of spirit drinking, or from a dislike to administer a poison which has itself caused the disease. This for a long time caused me to use this mode of treatment with extreme reluctance, and to restrict its employment to those cases in which it seemed indispensably necessary, but the results were so incontestible, and the diminution of the mortality so evident that I could not avoid adopting a method which has hitherto insured the safety of the patient, under circumstances in which it would otherwise have been placed in extreme hazard. If the probability of a recurrence of the disease were increased, it would still be a matter of doubt whether a physician should hesitate; but there is no reason for believing that there is an increased danger of a relapse. At least the examination of the register of the hospital proves that such is not the case. If the patient return to his former associates before the disease is completely passed, that is, before the restlessness is removed, a relapse into habits of intemperance is almost immediate; but if all remains of the disease be completely removed, the remembrance of the attack and its accompanying horrors, will in general preserve the patient, for a time, from a renewal of his vicious habits. A complete abandonment of them is unfortunately not common, and is scarcely practicable without an entire abstinence from intoxicating drinks.

Although the treatment of the disease by alcoholic stimulants is so universally successful, something more is required to insure their favourable results; that is, a judicious management of the complications which are so frequent in the disorder. Their treatment in general does not interfere with that of the delirium tremens, for in drunkards the constant habit of stimulation renders the system almost insensible to ordinary excitants. If the complication be inflammatory, venesection is occasionally advisable, but free local depletion by cupping, and the application of counter-irritants are absolutely necessary. This is particularly the case with the inflammations of the thorax and brain; if the latter organ be attacked, revulsive foot baths and cold applications to the head should be constantly kept up. Although an active congestion of the brain, which requires these antiphlogistic means, does not constitute an insuperable objection to the use of stimulants, these remedies should always be given in the least possible dose, and be immediately discontinued if the symptoms of the delirium tremens abate. If there is gastritis, ice and iced water should be given, and the stimulant discontinued as soon as possible. Many of our cases were of this complicated kind, but the mortality was not increased by them. Still the gravity of the prognosis is much increased, and they must occasionally prove fatal.

In recommending the stimulant treatment as on the whole the safest, especially in cases which cannot be carefully watched, I do not mean to assert that other methods of treatment will not be equally successful with extraordinary care, but I am fully convinced that no method is so safe and so practicable both in severe and in slight cases, especially if they cannot receive more than the ordinary attention which a physician in large practice can give them. But a physician, who is convinced of the propriety of the course, may leave a mild case to itself with the certainty that it will wear itself out, or he may adopt some other form of practice. Of these, the two which are most used in this city are the methods by opiates and by emetics.

As to the former, notwithstanding the predilection I once had for it, I would restrict the use of opiates to two different circumstances; one is the cases of delirium tremens which follow surgical accidents, in which the attack is often slight, but where rest is essential. In these cases I would at present give opium with stimulants, either the anti-spasmodics or the alcoholic stimulants. But the opium should be given in moderate doses, as thirty or forty drops of laudanum, repeated at intervals of two, three, or four hours. There is another case in which opium is useful, though I have not found it strictly necessary; that is, at the close of the disease, when the symptoms have subsided, either naturally or by the aid of treatment, but the patient remains restless and sleeps badly; a single dose of 10 or 12 grains of Dover's powder, or 25 or 30 drops of laudanum, given at night, will then often hasten the recovery. There are also times during the course of the disease in which the patient is disposed to sleep, but composes himself with difficulty, in which the same treatment will produce good effects. I do not object to opium given in this way to assist nature, I object only to relying upon it as the remedy by which sleep may be forced, to use a common expression.

The emetic treatment is in like manner often a successful one, and in the milder cases of the disease which occur after a forced abstinence from the habitual use of spirits, it is found to be successful. It was much relied upon by Dr. Klapp of this city, who contributed greatly to its introduction. The explanation which I give of the action of emetics, differs from that admitted by Dr. Klapp, who supposes that they act chiefly upon the stomach. It is their general, not their local action, which seems to me the most beneficial; the languor and relaxation which follows vomiting, calms the nervous agitation and disposes to a healthful sleep. If the vegetable emetics be prescribed, there is little danger following their use; but in patients who have had the facilities of indulging freely in large quantities of ardent spirits, that is, the drunkards belonging to those classes of society who are in comparatively easy circumstances, these remedies are attended with danger, and I have not found, as a general rule, that they are equally certain of producing a speedy cure as the stimulant practice.

In recommending alcoholic stimulants as the most certain remedy, I do not exclude anti-spasmodics and the different stimulants. These may be substituted in many cases for the former remedies, or they may be given in combination with them. The best are Hoffman's anodyne, assafœtida either in milk or tincture, capsicum and valerian. The simple infusion of valerian is an excellent calmant in these cases. Capsicum is best fitted to those cases in which there is much nausea with a sensation of sinking at the stomach, while there are few or no evidences of inflammation. It may be given in pills of one or two grains repeated every two hours, or in a larger quantity if the article be of feeble strength. Tonics are also of great benefit in many stages of the disease.

In the treatment which I have recommended, I am aware that I do not agree with some of my professional brethren, for whom I entertain the highest respect. In common with them I had my own repugnance for it to overcome; but the evidence of its effects has been of a nature, which I could not refuse to admit. I lay no claim, of course, to originality in advising a method which has been long used, other than what results from settling its value upon a more definite basis than has yet been done. It is possible that other methods may prove equally successful; for the disease tends to a natural recovery, and this termination may be favoured in many ways, but the results which I have given for the past year are upon so large a scale, that they clearly prove that no method can yield a more successful result.

